# Ethanol Metabolism in the Liver, the Induction of Oxidant Stress, and the Antioxidant Defense System

**DOI:** 10.3390/antiox11071258

**Published:** 2022-06-26

**Authors:** Martha Lucinda Contreras-Zentella, Daniel Villalobos-García, Rolando Hernández-Muñoz

**Affiliations:** Departamento de Biología Celular y Desarrollo, Instituto de Fisiología Celular, Universidad Nacional Autónoma de México (UNAM), Mexico City 04510, Mexico; mcontre@ifc.unam.mx (M.L.C.-Z.); aurum@ciencias.unam.mx (D.V.-G.)

**Keywords:** alcohol dehydrogenase, catalase, microsomal ethanol oxidation system, oxidant stress, alimentary status, alcohol liver disease

## Abstract

The liver metabolizes ethanol through three enzymatic pathways: alcohol dehydrogenase (ADH), cytochrome p450 (also called MEOS), and catalase. Alcohol dehydrogenase class I (ADH1) is considered the most important enzyme for the metabolism of ethanol, MEOS and catalase (CAT) are considered minor alternative pathways. However, contradicting experiments suggest that the non-ADH1 pathway may have a greater relevance for the metabolism of ethanol than previously thought. In some conditions, ethanol is predominately metabolized to acetaldehyde via cytochrome P450 family 2 (CYP2E1), which is involved in the generation of reactive oxygen species (ROS), mainly through electron leakage to oxygen to form the superoxide (O_2_^•−^) radical or in catalyzed lipid peroxidation. The CAT activity can also participate in the ethanol metabolism that produces ROS via ethanol directly reacting with the CAT-H_2_O_2_ complex, producing acetaldehyde and water and depending on the H_2_O_2_ availability, which is the rate-limiting component in ethanol peroxidation. We have shown that CAT actively participates in lactate-stimulated liver ethanol oxidation, where the addition of lactate generates H_2_O_2_, which is used by CAT to oxidize ethanol to acetaldehyde. Therefore, besides its known role as a catalytic antioxidant component, the primary role of CAT could be to function in the metabolism of xenobiotics in the liver.

## 1. Introduction

The liver metabolizes ethanol through three enzymatic pathways: alcohol dehydrogenase (ADH), cytochrome p450 (mainly isoform 2E1; also called MEOS (the microsomal ethanol-oxidizing system)), and catalase [1]. The reactions of each enzyme are shown below: 
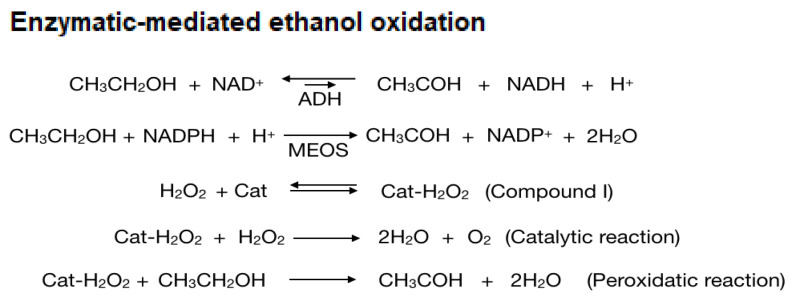


Alcohol dehydrogenase class I (ADH1) is considered the most important enzyme for the metabolism of ethanol [1,2,3,4]. MEOS and catalase (CAT) are considered minor alternative pathways [1,4,5], whereas the other classes of ADH (II, III, and IV) are mostly considered too inefficient to be relevant [1,2,6]. This is mainly due to the observation that fomepizole, a potent inhibitor of ADH1, inhibits around 80% of the rate of ethanol metabolism [7], whereas the remaining 20% is metabolized by the non-ADH1 pathway. There have been, however, contradicting experiments suggesting that the non-ADH1 pathway may have more relevance in the metabolism of ethanol.

**Figure 1:**
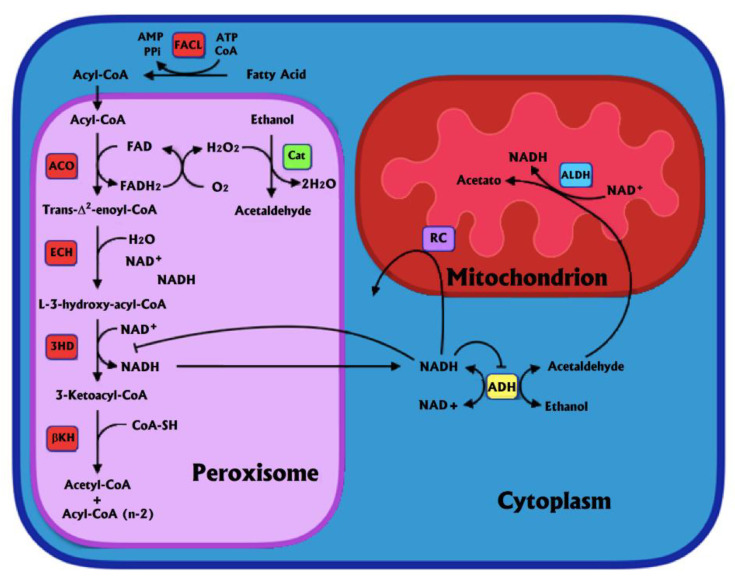
Redox interaction between ADH and peroxisomal ß-oxidation. Fatty acids are converted to Acyl-CoA and are transported into peroxisomes. During ß-oxidation, Acyl-CoA is oxidized, generating both H_2_O_2_ and NADH, with the former being reduced to H_2_O by catalase in the peroxidatic reaction. NADH is also generated from the oxidation of ethanol by ADH in the cytoplasm and of acetaldehyde by ALDH in the mitochondria. Since NADH inhibits both ADH and 3HD, its production from both enzymes directly reduces their activity. ADH, alcohol dehydrogenase; Cat, catalase; RC, respiratory chain; ALDH, aldehyde dehydrogenase; FACL, fatty acyl-CoA ligase; *ACO*, acyl-CoA oxidase; ECH, enoyl-CoA hydratase; 3HD, 3-hydoxyacyl-CoA dehydrogenase; ßKT, ß-ketothiolase.

From the late 1970s to the early 1990s, new ideas and models brought about new information that threw the paradigm into question, showing convincingly that ADH1 may not be as important as previously thought. After this period, interest in the ADH1-independent metabolism of ethanol rapidly waned, leaving the controversy without conclusion. To this day, there exists uncertainty as to how important the ADH1-independent pathway really is. Despite this, it is common for the modern literature to declare is as a minor or even irrelevant pathways (Figure 1).

However, it is known that the activity of CYP2E1 (which can generate significant amounts of reactive oxygen species) can be significantly induced due to chronic alcohol consumption, potentially leading to increases in cellular injury, lipid peroxidation, and oxidative and mitochondrial damage, and contributing to pathological processes [8,9].

Reactive oxygen species (ROS) are efficiently eliminated by antioxidant defense systems, which involve enzymes that detoxify oxygen free radicals, such as CAT, glutathione peroxidase (GSH-Px), and superoxide dismutase (SOD), all under normal physiological conditions [10]. Indeed, SOD can promote the conversion of superoxide radicals to hydrogen peroxide, and the latter (H_2_O_2_) is decomposed into water and oxygen by GSH-Px and CAT, preventing the accumulation of ROS [11]. CAT is one of the most important antioxidant enzymes in almost all aerobic organisms, where it breaks down two hydrogen peroxide molecules into one oxygen molecule [12] and two water molecules in a two-step reaction [13]. The first step of the reaction involves the formation of a distinct intermediate known as compound I (which is a covalent oxyferryl species having a porphyrin-cation radical) through the reduction of one hydrogen peroxide molecule [14,15]. In the second reaction step, compound I is reduced through redox reactions by a two-electron transfer from an electron donor (the second molecule of hydrogen peroxide), producing the free enzyme, O_2_, and water [13].

As previously mentioned, CAT can catalyze ethanol via oxidation to acetaldehyde [16], but it also exerts an antioxidant role in many tissues, including the heart [17]. The latter role is consistent with the ability of CAT to become overexpressed to protect against the ethanol-evoked dysfunction of myocytes isolated from male mice [18]. In contrast, we showed that an acute ethanol-evoked increase in cardiac CAT activity was associated with myocardial oxidative stress or dysfunction in endogenous estrogen-replete rats [19,20]. Although CAT protects against ethanol-evoked myocardial dysfunction, it has been reported that CAT inhibition by 3-amino-triazole (3-AT) causes cardiac oxidative stress and hypotension, revealing the antioxidant role of CAT played in the protection of myocardial cells [21]. In myocardial cells (cardiomyocytes), the increased expression of Nrf2 occurs simultaneously to that of SOD, CAT, and heme oxygenase-1 (HO-1), inhibiting the ethanol-induced overproduction of intracellular ROS [22] (Figure 1).

## 2. Ethanol Metabolism (Oxidation)

Inhibitors have been widely used to study the contribution of each metabolic pathway to ethanol oxidation; however, the lack of specific selectivity is a serious drawback. Fomepizole is a potent inhibitor of ADH1, and although it can also inhibit other classes of ADH, much higher concentrations are required to do so [23,24,25,26]. Hence, fomepizole is used as a selective inhibitor of ADH1. However, fomepizole also inhibits both CYP450 [27] and the production of H_2_O_2_, which decreases the catalase-mediated oxidation of ethanol [28].

Aminotriazole is an irreversible inhibitor of catalase, but it also inhibits CYP450 [29]. This inhibitor requires a high concentration and a relatively long time for complete inhibition to occur, and the presence of ethanol will completely protect CAT from inhibition [30]. Therefore, there has to be certainty that all of the CAT has been inhibited before the administration of ethanol, and this may be the reason for the variability seen with this inhibitor when it is administered in vivo to determine the importance of CAT [31], because CAT has such a high activity that a large portion of it has to be inhibited before a drop in alcohol oxidation can occur [32,33].

### 2.1. Methanol and Butanol as Specific Substrates

Contrary to human ADH or in other primates, the rodent enzyme is incapable of oxidizing methanol, but it is able to oxidize butanol [32]. Inhibitors have been widely used to study the contribution of each metabolic pathway to ethanol oxidation; however, the lack of total selectivity is a serious drawback. CAT is incapable of oxidizing butanol but it oxidizes methanol at the same rate as ethanol [34]. MEOS is able to oxidize all alcohols, having the most activity towards ethanol [35].

These properties allow methanol and butanol to substitute ethanol as a substrate for CAT and ADH, respectively. Since these substrates are specific for their respective enzymes, the rate of oxidation of methanol is indicative of the rate of ethanol oxidation in the absence of ADH, whereas the rate of oxidation of butanol is indicative of the rate of ethanol oxidation in the absence of catalase. Because methanol is not poisonous to rodents as it is to humans [36], it can be used in vivo.

### 2.2. Alcohol Metabolism in ADH− Deermice

In 1980, Burnett and Felder described a strain of deermouse lacking ADH1 (ADH−) that was capable of metabolizing ethanol [37]. Although humans have six different isoforms of ADH1 [1], rodents have only one [38]. Therefore, this mutant effectively lacks the only ADH considered important in ethanol metabolism (Figure 1).

ADH− deermice metabolize ethanol at 30 to 70% of the rates of deermice, with normal ADH (ADH+) activity in vivo [28,29,37,39,40,41]. With ADH1 out of the way, it became obvious that around half of the ethanol metabolized is independent of this enzyme. Hence, it became necessary to know which enzyme was responsible for the remaining metabolism of ethanol. Two main research groups emerged: the Lieber group tried to prove that MEOS was responsible, whereas the Thurman group tried to prove CAT was responsible. However, their experiments produced heavily contradicting results.

### 2.3. The Pro-CAT Approach

The rate of methanol metabolism in ADH− deermice closely follows (around 75%) that of ethanol, regardless of their molarity in blood [28,41,42], with similar results observed in perfused livers [28,41]. The in vivo metabolism of methanol is identical in ADH− and ADH+ deermice [27,28], which means the absence of ADH1 does not cause an increase in the metabolism of either alcohol through catalase.

When CAT is inhibited in vivo with aminotriazole in ADH− deermice, the ethanol oxidation rate diminishes by 75% [5,28,29]. Handler et al. [29] showed that 1.5 h after the administration of aminotriazole, the peroxidatic activity of CAT is reduced to less than 1% that of the control, whereas the activity of CYP450 (determined by aniline hydroxylation) is diminished by 80 to 90%. Six hours later, the rate of ethanol oxidation is completely restored. Simultaneously, the peroxidatic activity of CAT is almost completely restored, but the activity of CYP450 becomes undetectable, suggesting that the contribution of CYP450 to ethanol oxidation is negligible. The remaining 25% of the activity is presumably due to non-enzymatic routes, although the other classes of ADH may also participate (explaining the gap between methanol and ethanol metabolism in ADH− deermice).

Moreover, the butanol metabolism in perfused livers from ADH− deermice is low, whereas that of ethanol remains high [43], indicating that MEOS has little relevance. Since ADH1 is not present, what little butanol metabolism remains is most likely due to other classes of ADH or MEOS.

It is known that fructose stimulates the metabolism of ethanol by increasing the rate of oxidation of NADH (the rate-limiting step of ADH) [1]; this happens when perfused livers from ADH+ deermice are administered fructose while metabolizing ethanol [43].

However, when livers from ADH− deermice are used in the same experiment, the metabolism of ethanol is sharply reduced by fructose. In the same experiments, fructose slightly stimulated the metabolism of butanol in the livers from ADH− deermice. The same results were obtained in vivo, with fructose inhibiting the metabolism of ethanol in a dose-dependent manner [42]. In the same report, the methanol metabolism was shown to be inhibited in the same manner by fructose, indicating that ethanol and methanol are similarly metabolized (Figure 1).

### 2.4. The Pro-MEOS Approach

Contrasting experiments have shown that MEOS is primarily responsible for ethanol metabolism in ADH− deermice. Enzymes discriminate between isotopologues (the same substrate with a different isotopic composition), reacting more slowly with heavier isotopes. This isotope effect alters the Kcat/Km, and is specific for each isotope and enzyme. The isotope effect is the ratio of the rate constants for the reactions involving the light and heavy isotopically labeled substrates [40].

Damgaard [44] published a method to estimate the contribution of each pathway by determining the D (V/K) isotope effect (deuterium isotope effect on Vmax/Km) using deuterated ethanol. Using (1R)-[^2^H]ethanol, Takagi et al. [40] and Alderman et al. [29] showed that at low ethanol concentrations (5–10 mM) MEOS contributed almost 100% of the total in vivo ethanol metabolism in ADH− deermice, and around 35% in ADH+ deermice. Aminotriazole (1 g/kg) did little to modify the rate of ethanol metabolism in ADH+ deermice, whereas fomepizole (0.5 mmol/kg) completely abolished the ADH contribution. At high ethanol concentrations (40–70 mM), MEOS accounted for 100% of the rate of ethanol metabolism in ADH- deermice and 70% in the ADH+ strain.

The in vivo metabolism of butanol in ADH− deermice was shown to be 117µmol min^−1^ kg^−1^ at a blood concentration below 10 mM. This rate of elimination is comparable to that of ethanol metabolism in ADH− deermice at low concentrations (139 µmol min^−1^ kg^−1^). Since this strain of deermice lacks ADH1 and CAT cannot oxidize butanol, it was concluded that MEOS must be metabolizing it [45].

Furthermore, in hepatocytes from ADH− deermice, the rate of ethanol metabolism was inhibited in a dose-dependent manner by butanol [45,46], with similar results observed in vivo [47]. Additionally, the use of azide (a potent CAT inhibitor) did not modify the rate of ethanol metabolism in hepatocytes from naive and ethanol-fed ADH− deermice [45]. Since butanol is neither a substrate nor an inhibitor of catalase, these results strongly suggest that ethanol is not oxidized by this enzyme.

In vivo, fomepizole (0.5 mmol/kg) significantly decreased the metabolism of ethanol in ADH− deermice both at low (10 mM) and high (50 mM) ethanol concentrations, with decreases of around 20% and 40%, respectively [40]. Fomepizole does not inhibit catalase, but it inhibits the MEOS-dependent ethanol oxidation by more than 40% with 1 mM 4-methylpyrazole (4-MP) and 100 mM ethanol in vitro [27].

### 2.5. Production of H_2_O_2_

The oxidation of ethanol by CAT depends on the H_2_O_2_ production rate (relative to the CAT heme content) and the concentration of ethanol. Low H_2_O_2_ production rates and high ethanol concentrations give a higher proportion of peroxidatic (ethanol elimination) reaction, whereas high rates of H_2_O_2_ production and low ethanol concentrations give a higher proportion of catalytic (peroxide elimination) reaction. If the ratio of H_2_O_2_ production to the CAT heme concentration is sufficiently low, the decomposition of H_2_O_2_ will be almost completely peroxidatic, meaning that the rate of oxidation of ethanol will depend almost completely on the rate of H_2_O_2_ production [33]. The H_2_O_2_ production rate in the perfused liver was determined by the ability to directly observe CAT compound I in the perfused liver via dual-wavelength spectrophotometry [48] (Figure 1).

Early in vitro experiments showed that H_2_O_2_ production in the perfused liver was below 10 µmol g^−1^ h^−1^, more than an order of magnitude lower than the rate of ethanol oxidation, concluding that less than 10% of the total rate of ethanol oxidation was due to CAT [49].

Peroxisomal ß-oxidation (PßO) is a pathway that contrary to its mitochondrial counterpart, generates H_2_O_2_ through the enzyme acyl-CoA oxidase. The purpose of this pathway is not clear, but it has been hypothesized that long fatty acids (FFA) are first oxidized in peroxisomes, then after being shortened they are transported to mitochondria because the latter have trouble importing long FFA [50]. In the rat liver, around 25% of palmitate metabolism occurs in peroxisomes, where medium to long FFA (8-22 carbons) are shortened and then transported to mitochondria [51] (Figure 1).

Handler and Thurman [52] showed that in the presence of fomepizole, the addition of either 1 mM oleate, palmitate, or laurate to the perfusion medium increases the production of H_2_O_2_ from 15 to 61, 75, and 81 µmol g^−1^ h^−1^, respectively. These rates of H_2_O_2_ production are enough for the catalase-dependent oxidation of ethanol to rival that of ADH. Other experiments using different FFA have shown similar results [53,54]. As mentioned earlier, fructose decreases the metabolism of methanol and ethanol in ADH− deermice [42,43]. Experiments using perfused livers from rats showed that when butanol and methanol are administered simultaneously, fructose decreases the metabolism of methanol while increasing that of butanol [55], similarly to that observed in deermice. This happens because fructose depletes the hepatocyte’s ATP [42,55], which is needed to activate FFA to initiate ß-oxidation.

The concentration and composition of FFA in the blood can vary widely, depending on the diet and the alimentary status [56]. This matters because the H_2_O_2_ production rate depends on the type and concentration of FFA (being maximal at 1 mM in the rat liver) [52,53,54]. As known, the uptake of FFA by the liver increases during fasting [57], so it can be assumed that the H_2_O_2_ generated in the perfused liver is similar to that being generated in the liver in vivo. Moreover, other organs show considerable PßO, mainly the kidneys and heart, revealing an activity per unit of weight comparable to that of the liver [58,59]. The skeletal muscle has approximately one-tenth of the activity found in the liver per unit of weight in the fasted state [58], but because the total mass of skeletal muscle is much larger than that of the liver, it could be a very important source of H_2_O_2_, meaning it might be relevant in the oxidation of ethanol in vivo. It is worth noting that albumin (a fatty acid carrier) has to be present at a physiological concentration of about 4% for the liver to take up the fatty acids and increase H_2_O_2_ production. When used at a concentration of 1.7% (as well as lower concentration of fatty acids), it appears to be the reason for the initial H_2_O_2_ production of 10 µmol g^−1^ h^−1^ [31] (Figure 1).

## 3. Alimentary Status

Fasted individuals metabolize ethanol at a 25% lower rate than fed individuals. The amount of ADH in the liver, as well as the components of the malate–aspartate shuttle, is diminished during fasting, which has been proposed as an explanation for the reduced metabolism [1].

A redox interaction between ADH and PßO has been observed, in which the production of NADH by both pathways inhibits each other (Figure 1). PßO generates NADH, which has to be exported to the cytoplasm to be oxidized. This increase in cytoplasmic NADH diminishes the activity of ADH. Simultaneously, the production of NADH by ADH also decreases the rate of PßO, apparently inhibiting the NAD^+^-requiring ß-hydroxyacyl-CoA dehydrogenase [55]. Accordingly, in hepatocytes from fasted rats, 10 mM ethanol inhibits ß-oxidation of 1.3 mM palmitate by 20 to 25%, although it is not known how much corresponds to either mitochondrial or peroxisomal ß-oxidation [60]. In this regard, the relevance of CAT, as observed in ADH- deermice, as well as in experiments in which fomepizole was used, is most likely overestimated, since in these experiments there no NADH was produced from ADH that inhibited PßO.

Handler and Thurman [61] showed that in perfused livers from fed rats administered simultaneously with butanol (15–20 mM) and methanol (25–30 mM) and with independently measured uptake, the livers metabolized total alcohol at around 100 µmol g^−1^ h^−1^, from which more than 85% was accounted for by butanol. The administration of 1 mM oleate decreased the uptake of total alcohol by 15%, with the uptake of butanol (indicative of ADH activity) decreasing by 20%, whereas the uptake of methanol (indicative of CAT activity) was not significantly changed. In livers from fasted rats, the total uptake of alcohol was 85 µmol g^−1^ h^−1^, and the administration of oleate decreased the uptake of butanol by 60% (from 75 to 25 µmol g^−1^ h^−1^), while simultaneously increasing the uptake of methanol by 400% (from 10 to about 40 µmol g^−1^ h^−1^). The increase in CAT activity is insufficient to cover the loss of ADH activity, which causes a net decrease of 25% in the total rate of alcohol oxidation. Because butanol is an NADH generator, the partial inhibition of PßO by ADH was included in this experiment. It is possible that the decrease in ethanol oxidation observed in fasted individuals is due to increased ß-oxidation rather than or in combination with diminished ADH and malate–aspartate shuttle components.

This experiment suggests that CAT may become the most relevant enzyme for ethanol metabolism in the fasted state, in part because the concentration of circulating FFAs is increased and the rate of ß-oxidation is high in the liver, but also because with the highly diminished ADH activity, CAT takes on a greater proportion of the overall ethanol oxidation. The concentration (as a whole) of FFAs in the blood of fasted rats is typically in the range of 500 to 1000 µM [62]. In humans, this value is similar in the fasted state [56,63], meaning that the stimulation of H_2_O_2_ generation by fatty acids seen in the perfused liver may occur in vivo; however, it is not known whether CAT actually takes on such a large proportion of ethanol metabolism in the fasted state in vivo.

The expression of CYP2E1 increases during fasting [1,64]. We were unable to find information regarding how fasting changed the activity of MEOS; however, Chung et al. [65] concluded that fasting alone was insufficient to cause an increased expression of CYP2E1. They found that coprophagy resulting from fasting was the actual cause of the increased CYP2E1 expression, which may mean that the activity of MEOS is not changed by the alimentary status (Figure 1).

## 4. Alcohol Concentration

When a person consumes alcohol, the blood ethanol concentration can range from as low as 1 mM, where no effects are noticed, to above 100 mM, which is highly lethal [66,67]. Since ADH1 has a low Km for ethanol (around 1 mM in rodents, Table 1) [23], it would be expected to oxidize ethanol practically at a constant rate at concentrations above 10 mM. Contrary to this, multiple studies have come to the conclusion that ADH1 becomes increasingly inhibited by high ethanol concentrations [68,69,70,71,72,73]. Takagi et al. [73] showed that the in vitro activity of ADH from deermice at 25 mM ethanol is only 80% that of 5 mM ethanol, while at 50 mM ethanol it is 65% and at 100 mM it is 50%. Despite this, the rate of ethanol metabolism both in vitro and in vivo does not decrease at high ethanol concentrations; in fact, the opposite usually occurs.

At low concentrations of ethanol (below 20 mM), the rate of ethanol metabolism has been shown to be constant, as long as it does not get close to 1 mM [74]. However, both in vitro and in vivo experiments have shown that at high ethanol concentrations (above 20 mM), the rate stops being linear, with higher concentrations yielding larger increases. The increase varies widely between individuals of the same species, ranging from no increase whatsoever to above 50%, and it has been confirmed in rats, deermice, baboons, and humans [39,73,74,75,76], although it might not exist in mice, in which ethanol metabolism may decrease at high concentrations [77,78,79,80]. Since ADH1 is already saturated at such concentrations, a different enzyme is expected to metabolize the additional ethanol. Indeed, this increase in metabolism is insensitive to fomepizole and also occurs in ADH- deermice [73,81].

CAT catalyzes both the peroxidatic and catalytic reactions, and as previously stated these reactions compete with each other and their proportion depends on the H_2_O_2_ production rate, as well as the concentration of ethanol. In this regard, at any given rate of H_2_O_2_ production the peroxidatic reaction will never be 100% efficient, meaning that increasing the concentration of ethanol will always increase the proportion of the peroxidatic reaction, even if only by a small amount [33]. Therefore, it is possible that the increase in ethanol metabolism seen at higher concentrations stems, at least in part, from an increased peroxidatic reaction, which would explain the increase in methanol metabolism seen at higher concentrations in both ADH+ and ADH- deermice. Wendell and Thurman [75] showed that part of the increase at high ethanol concentrations is sensitive to aminotriazole in rats, suggesting that CAT is involved.

Since MEOS has a higher Km for ethanol (~10 mM for 2E1 in rats), this pathway would become more important at higher ethanol concentrations [77]. Aside from 2E1, there are other isozymes that contribute significantly to MEOS; perhaps most importantly the rat isozyme 1A2 has a Vmax towards ethanol more than twice that of 2E1, but it has a Km of 62 mM, and contrary to 2E1 it is insensitive to fomepizole [77]. This particular isozyme better explains the increase in ethanol metabolism seen at very high concentrations. In humans, there are at least six isozymes other than 2E1 capable of oxidizing ethanol at a significant rate, with varying activities and affinities towards ethanol, as well as varying liver contents [82].

## 5. Cyp2e1^(−/−)^ Mice

Vasiliou et al. [79] showed that female Cyp2e1^(−/−)^/Cs^b^/Cs^b^ mice (lacking CYP2E1 and having reduced CAT activity in the blood, brain, and kidneys, but not in the liver) [79,83] metabolized ethanol at 76% the rate of the wild-type strain, whereas its male counterpart showed no difference to its respective control. However, Cyp2e1^(−/−)^ mice did not show any difference in ethanol metabolism compared to the wild-type mice, regardless of the ethanol dose administered.

Furthermore, Kono et al. [84] showed that Cyp2e1^(−/−)^ mice metabolized ethanol at 3.7 ± 1.2 mmol kg^−1^ h^−1^, whereas the wild-type mice metabolized ethanol at 3.2 ± 0.4 mmol kg^−1^ h^−1^; however, after 4 weeks of ethanol treatment, the rates of ethanol metabolism increased to 4.9 ± 0.6 mmol kg^−1^ h^−1^ and 5.1 ± 0.8 mmol kg^−1^ h^−1^, respectively, revealing no difference between the groups. The authors demonstrated that the lack of CYP2E1 is insufficient to reduce the rate of ethanol metabolism in mice. Moreover, the latter jeopardizes the long-standing idea that MEOS is responsible for the increase in ethanol metabolism observed during chronic alcohol intake. To our knowledge, there are no studies regarding the metabolism of ethanol in knockout models for catalase.

## 6. Other Classes of ADH

While the roles of MEOS and CAT have been thoroughly studied, the roles of the other classes of ADH have been largely neglected [85]. Knockouts for classes I, III, and IV have been generated in mice, and in stark contrast with deermice, Adh1^(−/−)^ mice lose around 75% of the rate of ethanol metabolism [86]. ADH3 has a Km for ethanol above 1 M and a Kcat very similar to that of ADH1; accordingly, ADH3 has been thought to be unable to contribute to the metabolism of ethanol in any significant way. However, Haseba et al. [78] showed that if the hydrophobicity of the medium is increased with tert-butanol, the kcat/km of this enzyme increases 10-fold. Since the cytoplasm of hepatocytes is considerably more hydrophobic than the buffer solutions used to determine the activity of enzymes, the known Kcat/Km of ADH3 is most likely irrelevant in the intracellular context.

These authors showed that Adh3^−/−^ mice metabolized ethanol at a 2/3 rate of that of the wild-type mice at a dose of 4.5 g/kg (91 mM in blood), but at 2 g/kg (40 mM in blood) no difference was found. These results suggest that ADH3 becomes relevant at high ethanol concentrations; moreover, this enzyme appears to be more expressed during the chronic intake of alcohol, which has led to the possibility that this enzyme, and not MEOS, is responsible for the increased rate in ethanol metabolism seen in patients with alcoholism [85]. However, in an almost identical experiment, Deltour et al. [86] showed that Adh3^−/−^ mice metabolized ethanol at the same rate as wild-type mice, regardless of their blood concentration. They also showed that Adh4^−/−^ mice had an almost 30% reduction in the rate of ethanol metabolism. The reason for this discrepancy is unknown, but it must be clarified to understand the roles of both enzymes.

Furthermore, while Adh4^−/−^ mice showed no abnormalities, Adh3^−/−^ mice exhibited smaller litter sizes (6.95 vs. 8.47), lower postnatal survival (85% vs. 99%), and smaller body weights at 14 weeks of age (24.7g vs. 34.3g) than the wild-type mice [87]. These developmental alterations may be caused by a disruption in retinol metabolism, causing a deficiency of retinoic acid, which is a very important hormone that regulates development and gene expression. This makes sense, since ADH3 is expressed in low to moderate amounts throughout the body [23,24,88], whereas ADH4 is mostly confined to the stomach and digestive system [89]. The fact that the lack of ADH3 causes these issues means that the decreased ethanol metabolism observed by Haseba and Ohno [85] might be an artifact. There is also evidence that ADH2 may be important at moderate ethanol concentrations, perhaps contributing close to 30% at 22 mM ethanol due to its high expression in the liver [3,90].

Therefore, the idea that the liver metabolizes almost all the alcohol in vivo might be wrong. If ADH3 is indeed important, it could mean its relevance goes beyond the liver, since this enzyme is also expressed in every tissue in amounts very similar to those found in the liver [23,24,91]. Furthermore, PßO is significant in the kidneys and striated muscle, and since all tissues possess CAT, this could mean that ethanol might be significantly metabolized by extra-hepatic tissues (Figure 1).

Experiments using knockout models have not been conclusive, since they seem to contradict each other and other wild-type models. The reason for this may be caused by differences in experimental design (such as alimentary status, which most of the time is not specified) or by artifacts. Another possibility is that the metabolism of ethanol in a given species is inherently different from other species, meaning that trying to understand how rats metabolize ethanol by using knockout mice might be inadequate (not to mention humans, which are far more distantly related). This is illustrated by the observation that Adh1^−/−^ deermice metabolize ethanol at approximately 50% the rate of the wild-type mice, whereas in the case of Adh1^−/−^ mice this percentage drops to 25%. This detail in particular may explain the decrease in ethanol metabolism seen in mice at high concentrations, which makes sense given the importance of ADH1 in this particular species.

ADH1 is a very important enzyme for the metabolism of ethanol, perhaps being the most important in what could be considered “optimal conditions” (low ethanol concentration in the fed state). However, there is enough evidence to suggest that at high ethanol concentrations, as well as in the fasted state, MEOS, catalase, and ADH3 may become major ethanol-metabolizing pathways (Figure 1).

It seems that all classes of ADH, as a whole, are the main enzymes metabolizing ethanol in the fed state, with ADH2, ADH3, and ADH4 possibly making a significant contribution at higher concentrations, whereas CAT may become prominent in the fasted state. MEOS might be important at high ethanol concentrations and during alcoholism, but its role seems doubtful now, given that its contribution may have been confused with ADH3. Whether the non-ADH1 pathway is caused by CAT, MEOS, other classes of ADH, or a combination of them, it remains true that the metabolism of ethanol is far from being completely understood.

## 7. Ethanol-Induced Oxidants Stress 

**Figure 2:**
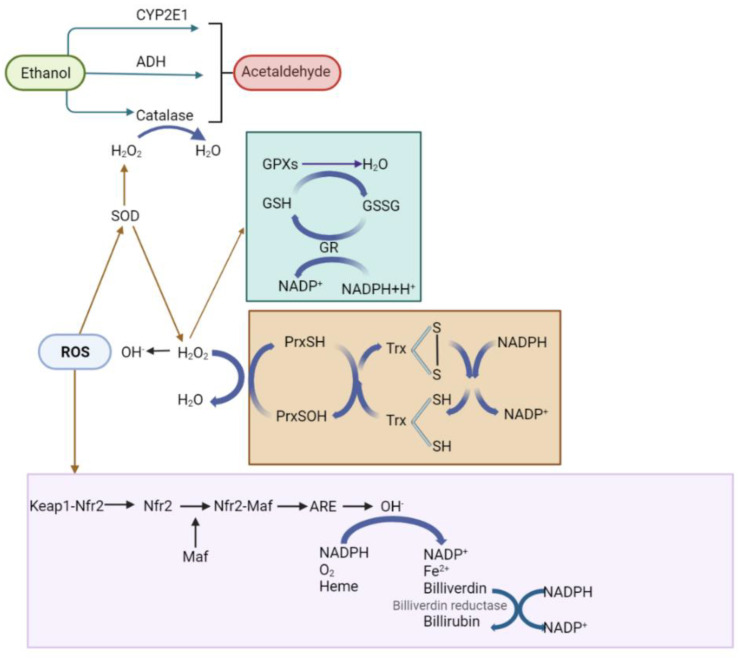
Ethanol oxidation, oxidant stress, and the antioxidant defense system. HO-1: Heme oxygenase-1, MAPK: Mitogen activating protein kinase; Nrf2: NF-E2-related factor-2, PKC: Protein kinase C, PP: Protein phosphatase, ROS: Reactive oxygen species, SOD: Superoxide dismutase, Keap1: Kelch-like ECH-associated protein 1, Trx: Thioredoxin, ARE: Antioxidant response element, ADH: Alcohol dehydrogenase.

Alcohol metabolism plays an important role in organ injury, particularly through the generation of ROS [92]. Hepatic ADH, along with CYP2E1 and CAT (including in other tissues such as the brain [92]), are major enzymes that catalyze ethanol oxidation to a highly reactive and toxic metabolite, acetaldehyde, whereas ethanol-evoked oxidative stress is implicated in heart injury [93]; this evidence is based on chronic exposure to ethanol (Figure 2).

Indeed, three mechanisms have been proposed to explain how alcohol causes liver injury, which, as previously mentioned, comprise acetaldehyde toxicity [94], the metabolic generation of ROS or exposure to oxidative stress [95,96], and the activation of an immune response that causes oxidative stress in hepatocytes [97,98]. Patients with alcoholic liver disease (ALD) seem to exhibit oxidative stress [99]; thus, increasing defense activities against this stress is important in the prevention of ALD.

Actually, during binge drinking, ethanol is predominately metabolized to acetaldehyde via the CYP2E1, which comprises a microsomal ethanol-oxidizing system that is involved in the generation of ROS [100,101]. Despite much evidence demonstrating a role for CYP2E1 in ALD, several studies have demonstrated that the consumption of ethanol-containing diets significantly increases hepatic CYP2E1 levels without significantly affecting potential “serum markers” for liver dysfunction and damage [102].

CYP2E1 has been shown to contribute to oxidative stress caused by alcohol, which is because the enzyme is a cytochrome reductase, which can leak electrons to oxygen to form the superoxide (O_2_^−^) radical or catalyze lipid peroxidation [103]. In fact, the induction of this enzyme by the chronic abuse of ethanol can increase the risk of liver damage by other agents. Furthermore, CYP2E1 can be induced in Kupffer cells, and macrophages overexpressing CYP2E1 have a more robust response to the putative ‘priming’ effect of alcohol [104] (Figure 2).

More recently, the advent of liver organoids has created a more accurate model for the in vitro study of hepatic metabolism [105]. It had previously been shown that in cultured cells overexpressing CYP2E1, mitochondrially localized CYP2E1 increases the generation of ROS in the presence of ethanol, giving more credence to the role of this enzyme in oxidative stress [106]. However, the cell culture is limited in its ability to mimic the inducible upregulation of CYP2E1 that is observed in vivo after ethanol consumption [107]. Angireddy et al. [107] showed that ethanol treatment of hepatic 3D organoids of HepaRG cells induced higher levels of CYP2E1 mRNA, which was reflected in a significant increase in CYP2E1 protein in the mitochondria. The increased mitochondrial CYP2E1 in turn induced liver toxicity by disrupting the activity of cytochrome c oxidase, inducing oxidative stress and depleting mtDNA. These studies further underline the significance of CYP2E1 in liver oxidative damage.

The superoxide radical is believed to play a central role in alcohol-induced liver injury, where exogenous SOD has a protective or antioxidant effect both in in vitro and in situ models of alcohol exposure. The genetic overexpression of either cytosolic Cu/Zn-SOD or mitochondrial Mn-SOD in liver cells has been shown to prevent alcohol-induced liver injury in rats fed alcohol [108,109]. The finding that both cytosolic and mitochondrial SOD isoforms were protective against alcohol-induced liver injury suggests that at least two distinct pools of O_2_^−^ production could be involved. On the other hand, a well-known radical that can be detected after alcohol exposure is the α-hydroxyethyl free radical. The formation of this radical depends on O_2_^−^ production [108,109]; however, O_2_^−^ is too weak of an oxidant to directly react with ethanol to form this product [110] (Figure 2).

Although O_2_^−^ is not a potent pro-oxidant per se, it appears to be a key initiator of oxidative stress during alcohol exposure. There is clear evidence that pro-oxidant formation is dependent on the production of O_2_^−^ during experimental ALD. However, the presence of this radical alone is not sufficient to explain the oxidative damage caused by alcohol (Figure 2).

The most obvious pathologic changes to the liver during alcohol exposure occur in hepatocytes, as evidenced by the accumulation of indices of oxidative stress (e.g., lipid peroxides), which are predominantly a hepatocellular event during alcohol administration. Therefore, hepatocellular oxidant production probably plays a key role in alcoholic liver injury. Besides the ethanol-inducible CYP2E1, mitochondria respiration and NAD(P)H oxidase (NOX) have been proposed as major sources of pro-oxidants in hepatocytes. In chronic liver disease, the activation of the NADPH oxidase isoforms leads to increased ROS generation and oxidative stress, resulting in inflammation and fibrogenesis. The activation of NADPH oxidase by hepatotoxic agents (e.g., bile salts) triggers cellular death pathways such as CD95-induced signaling to induce cell apoptosis [111]. Moreover, NOX isoforms found in hepatocytes participate in a variety of signal transduction cascades [112]. The elevated pro-oxidant production not only increases the net amount of pro-oxidants in the hepatocyte, but also directly damages mitochondrial proteins and DNA, which can aggravate mitochondrial aging and activate apoptotic pathways [113]. Furthermore, alcohol depletes mitochondrial GSH [114], resulting in increased hepatocellular apoptosis [115]. Therefore, hepatocellular pro-oxidant production is a crucial step in the progression of ALD.

Whereas CAT is considered a minor pathway of alcohol oxidation [116], a major function of this protein is as an antioxidant enzyme, eliminating ROS and maintaining redox balance. Interestingly, protein expression of CAT is decreased in samples from patients with non-alcoholic steatohepatitis (NASH). However, since the in vivo CAT activity relies on the physiological H_2_O_2_ concentration, the ex vivo quantitation may not precisely reflect the physiological CAT activity [9] (Figure 2).

## 8. Ethanol and Antioxidants

Humans consume many chemicals, including nutrients, phytochemicals, food additives, pharmaceuticals, and drugs. Whilst the intestine and liver absorb and metabolize many of them, some become more toxic when metabolized [117]. The metabolism of toxic compounds by the cellular enzymatic system is not fully efficient, and occasionally the metabolic processes generate products such as ROS that are more toxic than their original compounds [118].

CYP2E1 is a component of the cytochrome P450 system, which participates more specifically in the metabolism of xenobiotics, such as ethanol and FFA [119]. CYP2E1 is activated under diverse pathologies such as diabetes, obesity, starvation, cancer, alcohol liver disease, and non-alcoholic hepatic steatosis [120]. In addition, CYP2E1 directly promotes oxidative stress in mitochondria that is mediated by alcohol, damage in the mitochondrial DNA, and mitochondrial dysfunction in cells, and induces oxidative stress in the liver of ethanol-fed rats [121] (Figure 2).

**Figure 3:**
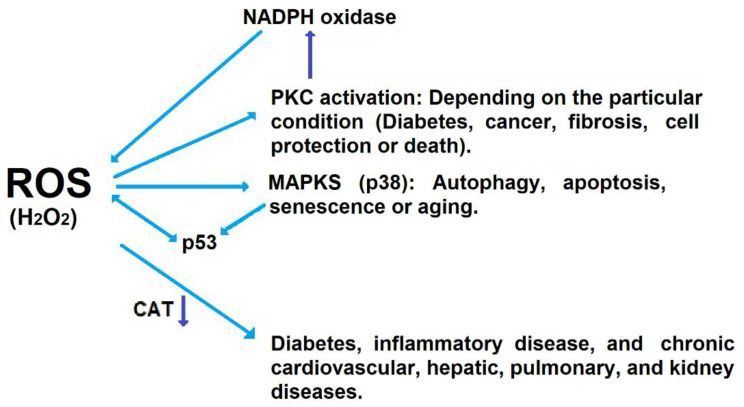
Signaling pathways activated by reactive oxygen species (ROS) and their involvement in pathological conditions. MAPK: Mitogen activating protein kinases, PKC: Protein kinase C, ROS: Reactive oxygen species, CAT: Catalase.

The toxic effects of the ingested ethanol can be explained by its metabolic products. Ethanol is mainly metabolized by the cytosolic alcohol dehydrogenase, which displays multiple isoenzymes and genetic polymorphisms. The other two oxidative pathways to oxidize alcohol and generate acetaldehyde are the inducible CYP2E1 system and the peroxisomal catalase, which are very important in specific conditions [122,123]. The produced acetaldehyde modifies the cellular function, depending on which enzymatic activities participate or acetaldehyde is formed. As a consequence of the differences in the ability to metabolize alcohol to acetaldehyde, it has been proposed that a high enzyme activity to metabolize alcohol makes people more susceptible to suffer alcohol-induced liver injury, in line with a higher level of acetaldehyde exposure, particularly in obese patients with excessive intake of alcohol. Furthermore, for acetaldehyde, alterations in the redox equilibrium during alcohol elimination may also be linked to other pathways and may stimulate effects leading to alcohol liver disease [124]. Therefore, the latter can cause an imbalance in the ROS levels, which is the main etiological factor for alcohol toxicity in the liver, causing a significant decrease in the levels of endogenous antioxidants such as GSH [125] (Figure 3).

In this regard, the cells have established a system to counterbalance the increase in ROS. This system comprises several types of cellular antioxidants [126], such as the enzymatic activities of SOD, catalase, glutathione peroxidase, and peroxiredoxins; hydrophilic antioxidants, such as glutathione, urate, flavonoids, and ascorbate; and lipophilic radical antioxidants, such as tocopherol, ubiquinol, and carotenoid. Antioxidants can also be categorized according to their source, including those that are endogenously synthesized such as enzymes and small molecules, as well as exogenous oxidants of foods such as minerals, carotenoids, phenols, vitamins, and flavonoids [99]; this system collectively reduces the oxidative state [118] (Figure 3).

In the cells of mammals, the greatest enzymatic antioxidants are SOD and CAT. In humans there are three types of SODs: CuZn-SOD, mitochondrial Mn-SOD, and extracellular SOD. SOD activity is induced in the cell as the first defense again free radicals of oxygen, transforming superoxide in oxygen and hydrogen peroxide [127]. The hydrogen peroxide can be formed and degraded in several subcellular compartments, and its local concentration is determined by its rates of synthesis, degradation, and diffusion, producing intracellular H_2_O_2_ gradients. The permeation of hydrogen peroxide across biological membranes involves specific channel proteins [128,129].

The hydrogen peroxide produced by SOD activity is disintegrated by catalase activity into molecular oxygen and water. Among ROS, H_2_O_2_ has been recognized as a second messenger, due to its reactions with specific protein cysteinyl residues in local environments that lower the pKa to afford specificity in time and space, which is needed during signaling [130,131]. Catalase is principally located in peroxisomes [132]. The participation of peroxisomes in several processes, such as cellular lipid metabolism, makes them very important in redox signaling networks; it has been proposed that the hepatic catalase decreases as liver alcohol disease worsens (Figure 2) [95,133,134].

Other antioxidant enzymes involved in the reduction of H_2_O_2_ are glutathione peroxidase, peroxiredoxins, and the Trx system (NADPH, thioredoxin reductase, and thioredoxin), which are often localized in several subcellular compartments [135,136]. The H_2_O_2_ is metabolically used by numerous peroxidases (Figure 2). These enzymes catalyze the oxidation of a wide number of organic and inorganic substrates by using the H_2_O_2_ in typically highly specialized reactions. Although the participation of these peroxidase reactions may be scarce, their activity is very significant metabolically in view of their temporal and spatial regulation. In proteins, the amino acid chain laterals most reactive to oxidization by H_2_O_2_ are cysteine, methionine, proline, histidine, and tryptophan.

The redox signaling started by H_2_O_2_ can progress via the direct oxidation of a protein, oxidation via a highly reactive sensor protein, activation of a target protein upon dissociation of an oxidized inhibitor, oxidation of a target protein via a secondary product generated through other antioxidants, inactivation of a scavenging protein to allow for the oxidation of the target protein, and association of the target protein with an H_2_O_2_-generating protein to allow site-directed oxidation. Furthermore, protein glutathionylation and other modifications can occur and serve in redox signaling [137]. Glutathione peroxidases are localized ubiquitously in the nucleus, plasma, cytosol, and mitochondria; they can also be associated with membranes [138]. Glutathione peroxidases have an antioxidant activity via which they produce alcohol or water from their organic hydroperoxide precursors using glutathione as a reducing agent [139]. Therefore, glutathiones coupled to catalase and SOD are vital to diminish the hepatic oxidative stress [140].

Besides the endogenous antioxidant mechanisms associated with enzyme and peptide activities, which have already been mentioned in this review, there are some exogenous antioxidants that have been reported to have an important action in relation to alcohol intake and liver damage. As results of animal tests, It has been proposed that several dietary supplements (vitamin A, vitamin E, carotenoids, vitamin B3, vitamin C, silymarin, curcumin, probiotics, zinc, S-adenosylmethionine, and garlic, in addition to antioxidants and anti-inflammatory agents), have very benefits effects in treating alcoholic disease [141].

### 8.1. Vitamins

These are natural compounds that could have antioxidant functions. For example, vitamins found in fruits or vegetables and some spices can modulate P450 enzymes such as CYPs. Indeed, alcohol intake causes a dysfunction in the metabolism of retinoids through several pathways: by inhibiting their oxidation by alcohol dehydrogenase; through the induction of CYP2E1, which participates in its metabolism; and by promoting the mobilization of the retinol out of the liver. As a consequence, it has been proposed that vitamin A may indirectly participate in the regulation of CYPs [142]. Moreover, a combination of L-ascorbic acid (vitamin C) with L-cysteine can induce a practically complete protection against acetaldehyde toxicity [143]. In fact, it has been proposed that vitamin C’s antioxidant activity avoids the progression of alcoholic disease to liver fibrosis, diminishing the oxidative stress, hepatic stellate cells activation, and cytotoxicity [138]. Additionally, in animal studies, it has been suggested that vitamin C can protect against injury to the mitochondrial membrane, DNA, and cell death as a consequence of its effects on enzymatic activity (such as glutathione transferase), ROS, and lipid peroxidation [144].

### 8.2. Antioxidants from Plant Extracts

It has been reported that polyphenols, which are antioxidants extracted from plants, can stimulate the previously mentioned endogenous antioxidant enzymes. This stimulating action of the phenol seems to be achieved by the participation of the Kelch-like ECH-associated protein (1-NF-E2)-related factor pathway and the antioxidant responsive elements [102].

Among phenols, resveratrol, a nonflavonoid phenol, is produced in some plants (grapes) in response to bacterial or fungal infection and has an antioxidant effect; resveratrol can decrease the production of free radicals implicated in hepatic damage [145]. Resveratrol is present in the red wine, and although alcohol intake in large amounts is associated with liver diseases and the production of oxidative stress, the moderate intake of red wine has been related with hepatoprotective, anti-inflammatory, and lipid-regulating effects, resulting from the polyphenolic compounds in red wine such as resveratrol [146,147]. In vitro, resveratrol increases HO-1 induction via the MAPK-Nrf2 pathway in PC12 cells [81]. However, the resveratrol concentration in red wine is not enough to prevent hepatic injury [102].

The other polyphenol compounds, namely the flavonoids, are largely present in flowers and fruits. Among these compounds, the anthocyanins are chemical compounds that give flowers a blue hue. These compounds increase Mn-SOD expression via extracellular-signal-regulated kinase (ERK) activation in HepG2 cells [148]. It has also been reported that an ethanol-induced acute gastric lesion was forestalled by the ingestion of strawberry extract, which is rich in anthocyanin, before ethanol treatment. This effect is associated with the induction of gastric antioxidant enzymes [149]. Fruits with high levels of anthocyanins belong to the Vaccinium genus, such as blueberry, cranberry, bilberry, Rubus berries (including black raspberry, red raspberry, and blackberry), blackcurrant, cherry, eggplant (aubergine) peel, black rice, Okinawan sweet potato, Concord grape, muscadine grape, red cabbage, and violet petals. Peaches and apples also contain anthocyanins [149,150].

#### Mulberry Leaves

The mulberry tree (*Morus alba* L.) is a traditional Chinese herb that grows in China, Korea, and Japan. The leaves of this plant contain polyphenols with biological functions such as antioxidant, anti-inflammatory, antidiabetic, and antitumor actions. Earlier studies suggested that the mulberry leaf extract has hepatoprotective effects against chronic alcohol-induced liver injury. The mulberry leaves’ polyphenols decrease lipid accumulation in the liver (steatosis) by downregulating fatty acid synthase and acetyl-CoA carboxylase and by fostering the AMP-dependent protein kinase pathway. It has also been reported that mulberry leaves raise the peroxisome-proliferator-activated receptor-α; these leaves reduce chronic alcohol-induced liver oxidative stress; increase the expression of caveolin-1; and shut-down the epidermal growth factor receptor (EGFR), the signal transducer activator of transcription 3 (STAT3), and iNOS by diminishing alcohol-induced liver damage. Hence, it has been proposed that mulberry leaves can reverse the alcohol-induced liver injury [151].

### 8.3. Other Polyphenols and Ethanol Toxicity

Flavonols are polyphenols (flavonoids) found in a wide variety of fruits and vegetables; these phenols act as antioxidants through their effects on the CYP (P450) activity, inhibiting CYP2C93 and CYP3A4 [152]. Among flavonols, quercetin is abundant in plants; in animal models, quercetin enhances lipid metabolism and ameliorates liver damage induced by ethanol administration, stimulating antioxidant molecules such as GSH and reducing CYP2E1 synthesis and enzymatic activity in human hepatocytes [102].

Other polyphenols such as catechins (cocoa beans, tea, blueberries, and wine) and tannins (cacao beans, tea, wines (mainly red), fruits, juices, nuts, chocolate, legumes, and cereal grains) have antioxidant effects too. In animal studies, catechin and tannin-rich extracts from pecan nut shells stimulate the activities of antioxidant enzymes [102,153,154]. Regardless of their antioxidant activity, polyphenols also have a small pro-oxidant activity, which may increase MAPK and phosphatase activity or stability before oxidative stress caused by ROS [155,156]. It has been proposed that phosphatases activated by antioxidants could inhibit MAPK pathway activation. An antioxidant pre-treatment has been associated with the hyper-activation of MAPKs and the diminution of the damaging stimuli. These findings could suggest that ROS or MAPKs are crucial regulators of antioxidant enzyme induction [102].

### 8.4. Other Antioxidants Compounds

Honokiol, a lignan identified in Magnolia officinalis, restored the hepatic GSH content and SOD activity and reduced inflammatory cytokine levels in an hepatic injury animal model, whereas berberine, a benzyl isoquinoline alkaloid in the Coptis genus, raises the levels of GSH and PGC1a and normalized CYP2E1 expression in an animal model of alcohol administration [102].

#### 8.4.1. Garlic

Garlic contains enzymes such as alliinase and organosulfur compounds such as alliin and its derivatives. There are extensive studies about the effects of garlic on several pathologies, such as hypertension, hyperlipidemia, diabetes mellitus, rheumatic disease, common cold, arteriosclerosis, and cancer. Its activity as a hypolipidemic results from its role in decreasing the biosynthesis and enhancing the hydrolysis of triacylglycerols (by raising the lipase activity) [157]. In mouse and rat models of alcohol exposure, the administration of H_2_S donors, such as diallyl trisulfide, diallyl disulfide, garlic oil, or garlic polysaccharide, substantially restricted lipid accumulation and diminished tissue injury. It has also been observed that serum enzymes associated with liver metabolism, such as alanine aminotransferase and aspartate transaminase, decrease their activity after garlic treatment. Recently, it was reported that aldehyde dehydrogenase can be repressed in the rat liver by some sulfane sulfur species, such as garlic-derived allyl sulfides, which release H_2_S in the organism. For this reason, H_2_S has to be utilized for the therapy of alcohol abuse [157]. In addition, diallyl disulfide and garlic oil, compounds that contain sulfur, have been reported to diminish alcoholic hepatic damage by raising HO-1 levels across the Nrf2 pathway and enhancing the GSH levels in vivo and in vitro [102]. Onion powder consumption, which contains high levels of sulfide compounds and flavonols, has also been reported to reduce hepatic CYP2E1 levels in normal rats [102].

#### 8.4.2. Terpenoids

Maslinic acid, a triterpenoid rich in basil, brown mustard, and other plants, has been reported to avoid hepatic injury via acute ethanol toxicity. These data suggest that some types of terpenoids may improve the symptoms of alcoholic disease [102].

#### 8.4.3. Ginger

This is a plant of the Zingiberaceae family. It has been observed that ginger has a hepatoprotective effect against ethanol, carbon tetrachloride, and acetaminophen-induced hepatotoxicity. Some dehydrated equivalents of gingerols, the shogaols, have been studied in vitro to elucidate the anti-inflammatory effects of ginger. The shogaol-derived nanoparticle (GDN) has an effect on hepatocytes, avoiding alcohol injury by facilitating the stimulation of the nuclear factor erythroid 2-related factor 2 (Nrf2), controlling the expression of a group of genes related with the liver-detoxifying or antioxidant response, and repressing the production of ROS, which protects the liver. It has been shown in lipid knockout and knockin mice that GDN-containing shogaol participates in the stimulation of Nrf2 dependent on TLR4/TRIF [158].

#### 8.4.4. Cannabidiol

This constituent of cannabis that has no psychotomimetic effects does indeed have extensive and complex immunomodulatory, antioxidant, anxiolytic, and antiepileptic effects. It has been suggested that cannabidiol can be used therapeutically in liver injury and in altered cognitive function related with chronic alcohol consumption [159]. Several pharmaceutical presentations have been approved in many countries (Canada was the first one in 2005) for the therapy of multiple sclerosis spasticity. However, a large part of the pharmacological action of cannabidiol seems to be based on mechanisms that do not involve cannabinoid receptors [159].

Studies with male mice and rats subjected to treatment with excessive alcohol led to the proposal that cannabidiol can assist as a complementary treatment by decreasing the incentive and motivation to consume alcohol. It has also been reported that cannabidiol regulates the inflammatory processes in the liver, reducing the steatosis or fibrosis produced by excess alcohol ingestion. The steatosis generated by excess alcohol ingestion stimulates hepatic stellate cells, which increase the synthesis of type 1-collagen-producing hepatic fibrosis. In studies in hepatic cells of ethanol-fed rats and mice, cannabidiol activated an endoplasmic reticulum stress reaction, causing the selective death of stimulated hepatic stellate cells as a result of inositol activation and involving apoptosis via the signal-regulating kinase 1/c-Jun N-terminal kinase (IRE1/ASK1/JNK) [159].

In another study in mice, cannabidiol avoided the increase in seric aspartate aminotransferase and alanine aminotransferase, enzymes associated with liver damage, and substantially reduced the rise in hepatic triglycerides. Cannabidiol also increased autophagy in vitro and in vivo, diminished the oxidative stress generated by excess alcohol consumption, and avoided the activation of the c-Jun N-terminal kinases (JNK). Remarkably, cannabidiol did not have an effect on the stimulation of cytochrome P450 E21(CYP2E1). Furthermore, cannabidiol diminished hepatic neutrophil infiltration, the indicators of liver inflammation (TNF-α), and the expression of pro-inflammatory chemokines, and impaired the liver damage generated by prolonged ethanol consumption [160]. However, it must be mentioned that there are no available data on cannabidiol’s efficiency in animal models of continual alcohol consumption.

#### 8.4.5. Carnosine

This compound is the natural dipeptide β-alanyl-L-histidine, and is considered to have biological effects mainly as an effective antioxidant. Carnosine could be beneficial in different pathologies, and special attention has been paid to the use of carnosine in neurologic and mental diseases. Moreover, carnosine may also play a role in alcoholism-induced physiological disturbances, which are accompanied by the activation of free-radical processes and the onset of oxidative stress [161].

#### 8.4.6. Silymarin

The cyanobacterium Spirulina maxima or Arthrospira maxima has shown hepatoprotective effects in rats and other experimental models. The hypolipidemic and antioxidant effects of spirulina have been demonstrated in humans. In addition, spirulina is a source of b-carotene, a-tocopherol, and phycocyanin, molecules with antioxidant properties [162]. Silymarin is a natural compound derived from this cyanobacterium that contains at least seven flavonoid ligands and the flavonoid taxifolin. The hepatoprotective and antioxidant activities of silymarin are based on its ability to inhibit the free radicals that are produced by the metabolism of toxic substances such as ethanol, acetaminophen, and carbon tetrachloride. The generation of free radicals is known to damage cellular membranes and cause lipoperoxidation; silymarin enhances hepatic glutathione and may contribute to the antioxidant defense of the liver. It has also been shown that silymarin increases protein synthesis in hepatocytes by stimulating RNA polymerase I activity [163].

## 9. Liver CAT and Its Role in Ethanol Oxidation (Peroxidation)

The liver peroxisomal CAT is a tetrameric, heme-containing enzyme that converts H_2_O_2_ to oxygen and water [164]. CAT can also oxidize ethanol to acetaldehyde as the end product in an H_2_O_2_-dependent fashion, although this is not a key pathway for ethanol elimination [165]. An increase in catalase activity has been reported after chronic ethanol feeding [166], and the rat’s CAT promoter contains a peroxisome-proliferator-responsive element (PPRE), which means it can be induced by peroxisome proliferators (Figure 2) [167].

As aforementioned, alcohol metabolism takes place mainly in the liver via oxidative enzymatic pathways involving enzymes such as ADH, CAT, and MEOS, which produce acetaldehyde in the three pathways. Free radicals are generated extensively in each pathway, altering cellular redox homeostasis [168], whereas ALDH oxidizes acetaldehyde to acetate. Activated CYP2E1 could increase ROS generation, including superoxide anions and hydroxyl radicals, resulting in oxidative stress and cell death [169]. Moreover, liver peroxisomal activity contributes to ethanol oxidation and ethanol reacts with H_2_O_2_ catalyzed by CAT, producing acetaldehyde and water (Figure 1 and Figure 2). The NADPH is known to be tightly bound to mammalian CAT in order to offset the ability of CAT’s substrate (H_2_O_2_) to convert the enzyme to an inactive state (compound II), and indeed NADPH may protect CAT from oxidative damage through actions broader than merely preventing the formation of compound II [170]. The overall conversion of H_2_O_2_ to H_2_O and O_2_ requires that the enzyme (CAT) alternates between being ferri-CAT and compound I (Figure 2) [171]. Hence, the CAT activity relies on the cellular level of H_2_O_2_. The ability of CAT to metabolize ethanol can be increased under oxidative stress with an increase in cellular H_2_O_2_ production [172]. Therefore, under conditions of oxidant stress (and H_2_O_2_ production), CAT-mediated ethanol oxidation may be increased (Figure 2) [165]. The increased rate of methanol uptake observed in livers from fasted rats, in the presence of FFA, is due to the increased peroxidation of methanol via CAT-H_2_O_2_ [61]. In addition, interactions between the ADH and CAT pathways occur and are mediated via the elevation of the pyridine nucleotide redox state. Redox interactions between catalase and ADH suggest that the NAD/NADH redox state within the peroxisome and the cytosol are in equilibrium [55].

The CYP2E1 activity can be significantly induced by alcohol consumption and can cause ethanol-induced oxidative stress and mitochondrial toxicity leading to cell injury. In contrast, CAT is considered to be an antioxidant enzyme, playing a critical role in eliminating ROS and maintaining redox balance, and its protein expression is significantly decreased in non-alcoholic steato-hepatitis (NASH) samples, although the ex vivo CAT activity remains stable, indicating that ex vivo quantitation may not precisely reflect the physiological CAT activity [9].

CAT overexpression has been shown to protect against ethanol-evoked dysfunction of myocytes isolated from male mice; however, an acute ethanol-evoked increase in cardiac CAT activity has been associated with myocardial oxidative stress or dysfunction in endogenous estrogen (E2)-replenished rats [19]. Furthermore, CAT inhibition abolishes not only O_2_ production from H_2_O_2_ degradation, but also NOX2-dependent superoxide production in podocytes challenged with both H_2_O_2_ and ethanol. These data suggest that exogenous H_2_O_2_ does not induce oxidative stress due to rapid degradation to produce O_2_ in podocytes, but that the oxygenated podocytes become sensitive to acute ethanol challenge and undergo apoptosis via a TRPC6-dependent elevation of intracellular Ca^2+^ [173].

### 9.1. Role of CAT in Fructose- and Lactate-Induced Liver Ethanol Oxidation

Fructose has been recognized for a long time as a substance capable of significantly increasing the oxidation of ethanol both in vivo and in vitro, a phenomenon that has been named *the fructose effect* [174,175]. The exact mechanism by which fructose increases the oxidation of ethanol is, however, not well understood, as its metabolism involves several pathways (Figure 1). It has been thought that the metabolism of fructose increases NADH oxidation, which allows a faster dissociation of the ADH-NADH complex (the rate-limiting step of hepatic ethanol oxidation) [176,177]. Indeed, it has been observed that fructose decreases the rates of ethanol metabolism in livers from ADH− deermice, possibly by affecting either the CAT enzyme or the supply of H_2_O_2_, or both [43]; indeed, fructose decreased the rates of liver H_2_O_2_ generation [43].

On the other hand, lactate has been known to increase the oxidation of ethanol both in vivo and in vitro [5]. However, the oxidation of lactate to pyruvate through LDH generates NADH; thus, it should be expected to slow the metabolism of ethanol, because the produced NADH would inhibit ADH [5]. Lactate could increase the concentration of the components of the malate–aspartate shuttle, which increases the rate of transport of reducing equivalents to the mitochondrial matrix [178]. This could mean that increases in the ATP demand through gluconeogenesis would increase the rate of oxidation of NADH in the respiratory chain [179].

In our hands and using liver slices, we found that the addition of 10 mmol L^−1^ lactate increased the rate of ethanol oxidation2-fold. Here, 4-MP, an ADH inhibitor, drastically decreased the rate of ethanol oxidation but did not inhibit the stimulation due to lactate. In contrast, the catalase inhibitor, 3-AT, completely inhibited the stimulation. From these results, it appears that the acceleration of ethanol oxidation by lactate is completely independent from ADH, which contradicts previous hypotheses, since inhibiting ADH activity potentiates the ethanol oxidation by CAT [169].

### 9.2. Production of MDA by Liver Slices after Ethanol Oxidation

**Figure 4:**
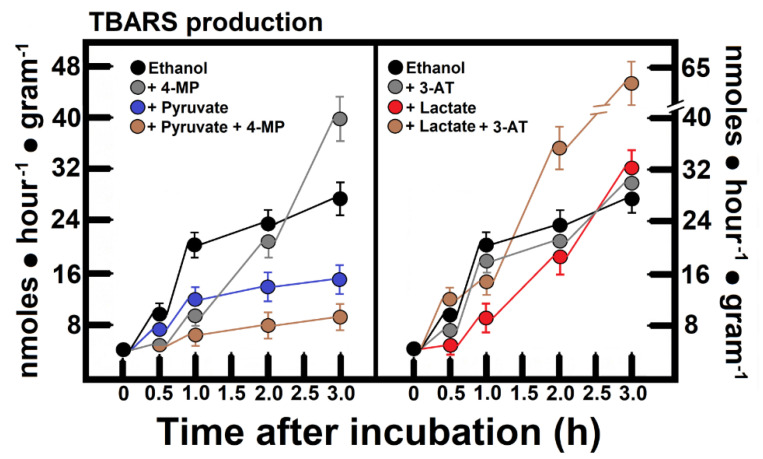
Production of TBARS by liver slices after ethanol oxidation in the presence of lactate or pyruvate. Results are the mean ± SE of 4 individual observations per experimental group. The left panel depicts TBARS concentration (mainly MDA) in the incubation medium after oxidation of 10 mmol L^−1^ ethanol, in the presence of pyruvate (30 mmol L^−1^) and 4-MP (1 mmol L^−1^). The right panel shows the same but with the addition of lactate (20 mmol L^−1^), as well as the effect exerted by 3-AT on the same parameter. Experimental groups are designated by symbols at the top of the figure.

Figure 4 depicts the rate of malondialdehyde (MDA) production (as the area under the curve, AUC) as a possible target of ethanol-induced H_2_O_2_ formation. The ethanol oxidation by liver slices was accompanied by the appearance of TBARS in the incubation medium (AUC = 14.1 ± 1.7 nmol h^−1^), which was increased when 4-MP was also added to the liver slice preparation (AUC = 20.1 ± 2.5 nmol h^−1^, *p* < 0.01 against controls). The addition of 30 mmol L^−1^ pyruvate exerted an interesting antioxidant effect on the ethanol-induced lipid peroxidation because the MDA release reached 7.7 ± 1.2 nmol h^−1^ (*p* < 0.01 vs. controls), an action that was potentiated even when ethanol oxidation was inhibited by 4-MP (AUC = 4.7 ± 0.7 nmol h^−1^, *p* < 0.01 against controls, Figure 4). The presence of 20 mmol L^−1^ lactate, despite this monocarboxylic acid significantly increasing ethanol catabolism, did not significantly modify the MDA formation (AUCs = 16.7 ± 2.8 vs. 14.1 ± 1.7 nmol h^−1^ of MDA, in controls; Figure 4). However, when the catalase activity was inhibited by 3AT in the presence of ethanol and lactate, these liver slices greatly increased the production and release of MDA against controls (AUCs = 25.8 ± 3.4 vs. 14.1 ± 1.7 nmol h^−1^ of MDA, in controls; *p* < 0.01, Figure 4), and even against lactate alone (AUCs = 25.8 ± 3.4 against 16.7 ± 2.8 nmol h^−1^ of MDA, with lactate alone; Figure 4). These results give further support to the statement that the addition of lactate generates H_2_O_2_, which is used by catalase to oxidize ethanol to acetaldehyde.

We found that the inhibition of ADH in the presence of ethanol increased the rate of lipid peroxidation (Figure 4), whereas pyruvate exerted an evident protective action, probably acting as an antioxidant. Pyruvate treatment may play a beneficial role in reducing vanadium-triggered health hazards [180]; moreover, sodium pyruvate added to red blood cells has a beneficial effect in alleviating liver injury after its transfusion in vivo [181]. Regarding lactate and ethanol oxidation, despite lactate increasing ethanol catabolism, it did not increase MDA formation, but when CAT activity was inhibited, there was a large increase in MDA production and release (Figure 4). Therefore, these results give further support to the statement that the addition of lactate generates H_2_O_2_, which is used by CAT to oxidize ethanol to acetaldehyde. Hence, lactate oxidase’s main function could be to maintain a stable supply of H_2_O_2_ as a way to keep the peroxidatic reaction active at all times. It is possible that the main role of CAT is to function in the metabolism of xenobiotics in the liver, where pro-oxidant reactions ought to be involved.

## 10. Conclusions

ADH1 is a very important enzyme for the metabolism of ethanol, perhaps being the most important in what could be considered “optimal conditions” (low ethanol concentration in the fed state). However, there is enough evidence to suggest that at high ethanol concentrations, as well as in the fasted state, MEOS, catalase, and ADH3 may become major ethanol-metabolizing pathways. It seems that all classes of ADH, as a whole, are the main enzymes metabolizing ethanol in the fed state, with ADH2, ADH3, and ADH4 possibly making significant contributions at higher concentrations, whereas catalase may become prominent in the fasted state. MEOS might be important at high ethanol concentrations and during alcoholism, but its role seems doubtful now, given that its contribution may have been confused with ADH3. Whether the non-ADH1 pathway is caused by CAT, MEOS, other classes of ADH, or their combination, it remains true that the metabolism of ethanol is far from being completely understood.

It has been proposed that one important mechanism underlying alcoholic liver injury is the metabolic generation of ROS or exposure to oxidative stress, especially through the participation of the CYP2E1, as its expression is increased after the chronic consumption of ethanol-containing diets. CYP2E1 has been shown to contribute to the oxidative stress caused by alcohol, since this enzyme is relatively coupled to a cytochrome reductase, which can leak electrons to oxygen to form the O_2_^•−^ radical or catalyze lipid peroxidation. Besides the endogenous antioxidant mechanisms, there are exogenous antioxidants that have been reported to have important actions on alcohol intake and liver damage, including several dietary supplements (vitamin A, vitamin E, carotenoids, vitamin B3, vitamin C, silymarin, curcumin, probiotics, zinc, S-adenosylmethionine, and garlic), such as having beneficial effects in treating alcoholic disease

CAT belongs to a group of antioxidant enzymes that detoxify oxygen-free radicals, and its action is involved in other functions, such as the regulation of the integrin pathway during proliferation or migration, among others. However, CAT activity can, in turn, generate pro-oxidant reactions, such as ethanol peroxidation. Despite the evidence that CAT can play a significant role in in vivo ethanol oxidation, it is still considered a minor pathway for alcohol metabolism. In this context, we have recently shown that CAT actively participates in the lactate-stimulated liver ethanol oxidation, where the addition of lactate generates H_2_O_2_, which is used by CAT to oxidize ethanol to acetaldehyde. The main function of lactate oxidase could be to maintain a stable supply of H_2_O_2_ as a way to keep the peroxidatic reaction active at all times. Therefore, it is possible that the main role of CAT is to function in the metabolism of xenobiotics in the liver.

## Figures and Tables

**Table 1:** Kinetic constants for rat liver ADH classes towards ethanol.

ADH	Km (mM)	Kcat (min^−1^)	Kcat/Km (min^−1^mM^−1^)	Ref
I	1.4	39	28	[9]
II	5000	1000	0.2	[9]
III	>3000	-	~0.001	[11]
IV	5000	2400	0.48	[11]

## Data Availability

Not applicable.
